# Is the Scale Up of Malaria Intervention Coverage Also Achieving Equity?

**DOI:** 10.1371/journal.pone.0008409

**Published:** 2009-12-22

**Authors:** Richard W. Steketee, Thomas P. Eisele

**Affiliations:** 1 Malaria Control and Evaluation Partnership in Africa (MACEPA)-PATH, Ferney-Voltaire, France; 2 Department of International Health and Development, Tulane University School of Public Health and Tropical Medicine, New Orleans, Louisana, United States of America; London School of Hygiene and Tropical Medicine, United Kingdom

## Abstract

**Background and Methods:**

Malaria in Africa is most severe in young children and pregnant women, particularly in rural and poor households. In many countries, malaria intervention coverage rates have increased as a result of scale up; but this may mask limited coverage in these highest-risk populations. Reports were reviewed from nationally representative surveys in African malaria-endemic countries from 2006 through 2008 to understand how reported intervention coverage rates reflect access by the most at-risk populations.

**Results:**

Reports were available from 27 Demographic and Health Surveys (DHSs), Multiple Indicator Cluster Surveys (MICSs), and Malaria Indicator Surveys (MISs) during this interval with data on household intervention coverage by urban or rural setting, wealth quintile, and sex. Household ownership of insecticide-treated mosquito nets (ITNs) varied from 5% to greater than 60%, and was equitable by urban/rural and wealth quintile status among 13 (52%) of 25 countries. Malaria treatment rates for febrile children under five years of age varied from less than 10% to greater than 70%, and while equitable coverage was achieved in 8 (30%) of 27 countries, rates were generally higher in urban and richest quintile households. Use of intermittent preventive treatment in pregnant women varied from 2% to more than 60%, and again tended to be higher in urban and richest quintile households. Across all countries, there were no significant male/female inequalities seen for children sleeping under ITNs or receiving antimalarial treatment for febrile illness. Parasitemia and anemia rates from eight national surveys showed predominance in poor and rural populations.

**Conclusions/Significance:**

Recent efforts to scale up malaria intervention coverage have achieved equity in some countries (especially with ITNs), but delivery methods in other countries are not addressing the most at-risk populations. As countries seek universal malaria intervention coverage, their delivery systems must reach the rural and poor populations; this is not a small task, but it has been achieved in some countries.

## Introduction

Malaria is not an equitably distributed infection or disease. Young children and pregnant women in rural and poor households in sub-Saharan Africa bear the brunt of malaria's morbidity and mortality [1]–[8].

As the Roll Back Malaria (RBM) effort set out to halve the malaria burden by 2010 [9], the early years from 1999 through 2004 were characterized by low coverage [10] and unequal distribution of prevention interventions whereby the poorest households had the lowest coverage [11], [12]. With more recent calls for Scale up for Impact (SUFI) [13], [14], malaria elimination [15], [16], and for universal coverage with malaria control interventions [17], addressing those at greatest risk of malaria has been in the forefront of discussions. The call for achieving universal coverage has focused initially on prevention interventions such as insecticide-treated mosquito nets (ITNs), indoor residual spraying (IRS), and prevention during pregnancy with ITNs and intermittent preventive treatment (IPTp). Achieving universal coverage among at-risk populations is warranted as prevention interventions have been shown to benefit even those not directly covered through a “community effect” when high population coverage is achieved [18]–[20]. Universal coverage would imply that everyone would have the needed preventive and curative interventions (100% and thus fully ‘equitable’). However, while *en route* to that goal, different intervention delivery strategies could lead to markedly variable coverage levels across the most at-risk populations [11], [21].

The malaria control community might see a dilemma here. As malaria is not an equitable disease, there is both an argument for targeting those at risk and an argument for providing universal population coverage (not specifically targeted) because of the additional community effect benefit. A possible resolution for the dilemma is to seek universal coverage and while achieving this, assuring and documenting that at least the same (equitable) coverage is attained for all populations at risk. In the context of the recent scale up of malaria interventions and the call for universal coverage in sub-Saharan Africa, we examined recent evidence from nationally representative household surveys to document whether national malaria programs have been achieving equity in the delivery of malaria prevention and treatment.

## Methods

### Definitions

Inequities in health are considered those differences that are “not only unnecessary and avoidable, but in addition, are considered unfair and unjust” [22]. Inequities are frequently considered on the basis of demographic and/or socioeconomic status, often measured in asset-based wealth quintiles [23], geography (country region, or urban versus rural dwelling), sex, age, and ethnicity. Recent nationally-representative population-based surveys have systematically collected information on wealth quintile, urban and rural dwelling and sex; all of which are considered here. Information from a given country may be available on ethnic or provincial or regional differences, but when examining across many national surveys, these characteristics are not comparable so are not considered here. Comparisons were examined specifically among those most vulnerable: children under the age of 5 years and pregnant women.

### Data Considered

Published reports were reviewed from recent nationally-representative household surveys conducted in sub-Saharan African from 2006 through 2008, a period following most recent intervention scale-up efforts, with the reports available by July 2009. In order to optimize the standard approach to data collection, only results from Demographic and Health Surveys (DHS: http://www.measuredhs.com/), UNICEF Multiple Indicator Cluster Surveys (MICS: http://www.unicef.org/statistics/index_24302.html) and RBM Partnership Malaria Indicator Surveys (MIS: http://www.rollbackmalaria.org/mechanisms/merg.html#MIS) were considered. The survey results are maintained on the UNICEF Child Info web site (www.childinfo.org) and include standardized information on household ownership and use of ITNs, use of prevention in pregnancy (IPTp and ITNs), and use of malaria treatment for children with recent fever illness. The surveys typically collect information on household location (urban or rural based on country definitions), child sex, and present household wealth quintiles based on a standard household asset index. Some surveys did not include full information on certain characteristics, thus the denominator of surveys varies for certain comparisons. While nationally-representative surveys have been conducted in malaria-endemic countries outside Africa and it is possible that more recent African national survey results may exist, surveys in more than one-half of the malaria-endemic sub-Saharan African countries were available and fitted our inclusion criteria. This group included a spectrum of low (less than 10%) to high (greater than 50%) coverage for the malaria interventions, and the information is summarized here.

### Measurements

The following malaria intervention outcome indicators consistent with the recommended RBM definitions were assessed [24]: the proportion of households with at least one ITN; the proportion of children under 5 years old with fever in the past 2 weeks who received an antimalarial: and the proportion of women of reproductive age who received at least two doses of sulfadoxine-pyrimethamine (SP) during their last pregnancy.

We assessed equity in intervention coverage with the following indicators: 1) urban versus rural status of household; 2) highest versus lowest wealth quintile; and 3) male versus female child gender. As there is no single standard for establishing a measure of equity for a given comparison (e.g., when comparing socioeconomic status across quintiles), we established an “equity index” as the ratio between intervention coverage between the two categories for each of the equity indicators outlined above. As such, an equity index greater than 1.0 suggests over representation of intervention coverage among urban households, the wealthiest households and male children.

Additionally, to assess whether malaria intervention coverage was statistically different between measures of equity, we performed a simple Pearson's Chi-square test to determine differences in these outcome indicators by urban versus rural status, highest versus lowest wealth quintile, and use among male versus female children. For these comparisons, the raw survey datasets were not used to ascertain Ch-square test statistics; instead, cells based on sample size and coverage estimates were used to create 2x2 tables. Where sample sizes were not available by wealth quintile (1 study), equal distribution across quintiles was assumed for calculating Chi-square test statistics. This approach does not account for the effect of clustered data as a result of the two-stage cluster sampling designs employed by the DHS, MICS and MIS, and thus the statistical tests used here may slightly overestimate statistical significance. However, we assert that this approach is sufficient for this description of how coverage outcomes differ by equity factors. In general, a coverage indicator was considered equitable if it achieved equal or higher coverage among poor and/or rural populations, with the probability of committing a type-1 error set at 0.05.

As multiple comparisons are presented in the figures, countries were grouped as equitable if the equity index for all variables considered was less than 1.2; countries where the equity index exceeded 1.2 for one or both comparisons were grouped as having inequitable distribution of the intervention coverage.

The prevalence of malaria parasite infections and moderate-severe anemia (Hb<8 g/dl) was assessed among children under 5 years old across eight national MIS's between 2006 and 2008. To show where morbidity was concentrated, differences in these morbidity outcomes were assessed by urban and rural status and poorest versus wealthiest quintiles using a Pearson's Chi-square test statistics, as described above.

## Results

**Figure 1:**
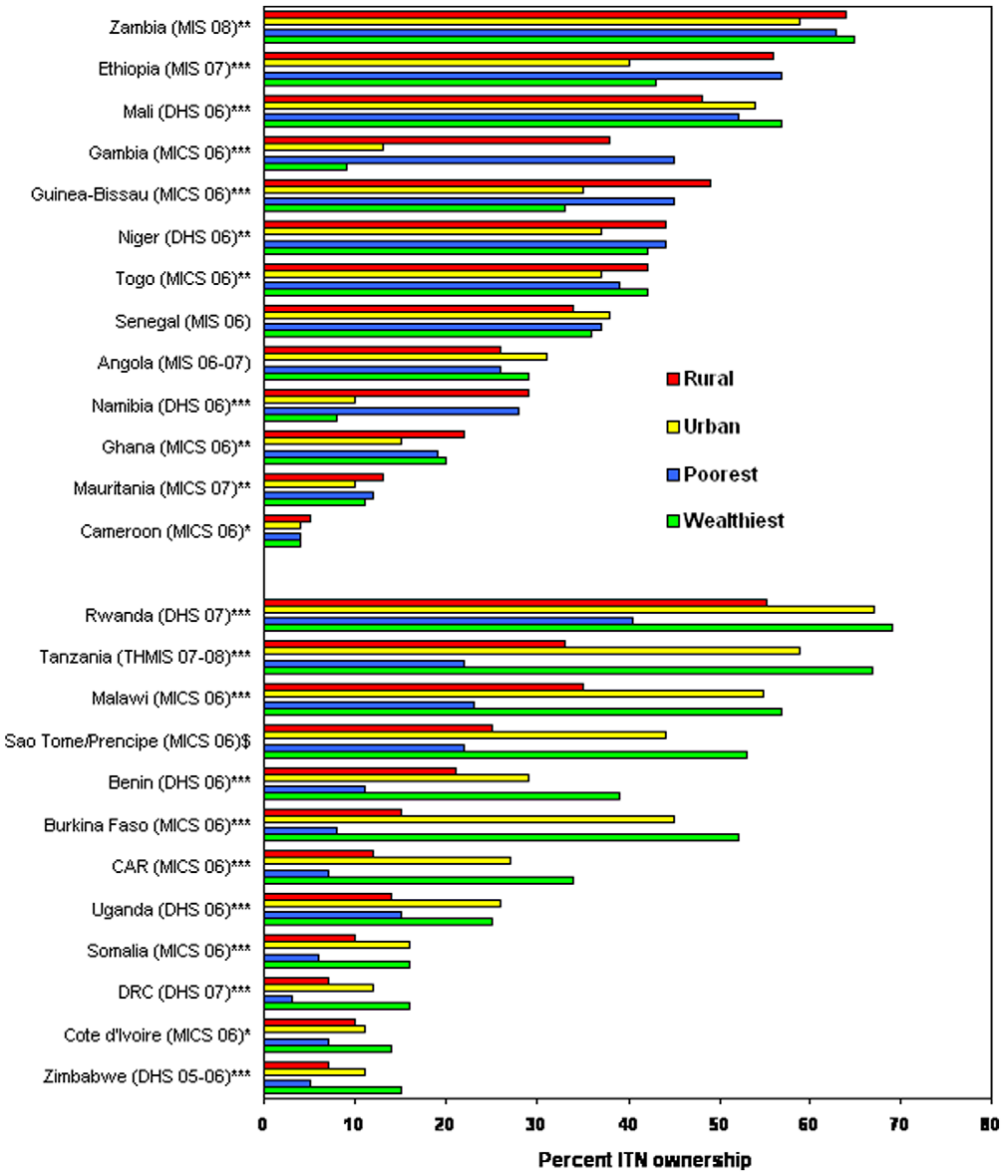
Equity in household ownership of ITNs. Percent household ownership of at least 1 ITN, by household residence and poorest versus wealthiest quintile, from national household surveys 2006–2008. Top group of countries are those achieving equity across rural-urban and wealth quintiles; bottom group are those not achieving equity across these categories. *Wealth statistically different (P-value<0.05); **Urban/rural statically different (P-value<0.05); ***Wealth and urban/rural statistically different (P-value<0.05); $Data not available for statistical test.

**Figure 2:**
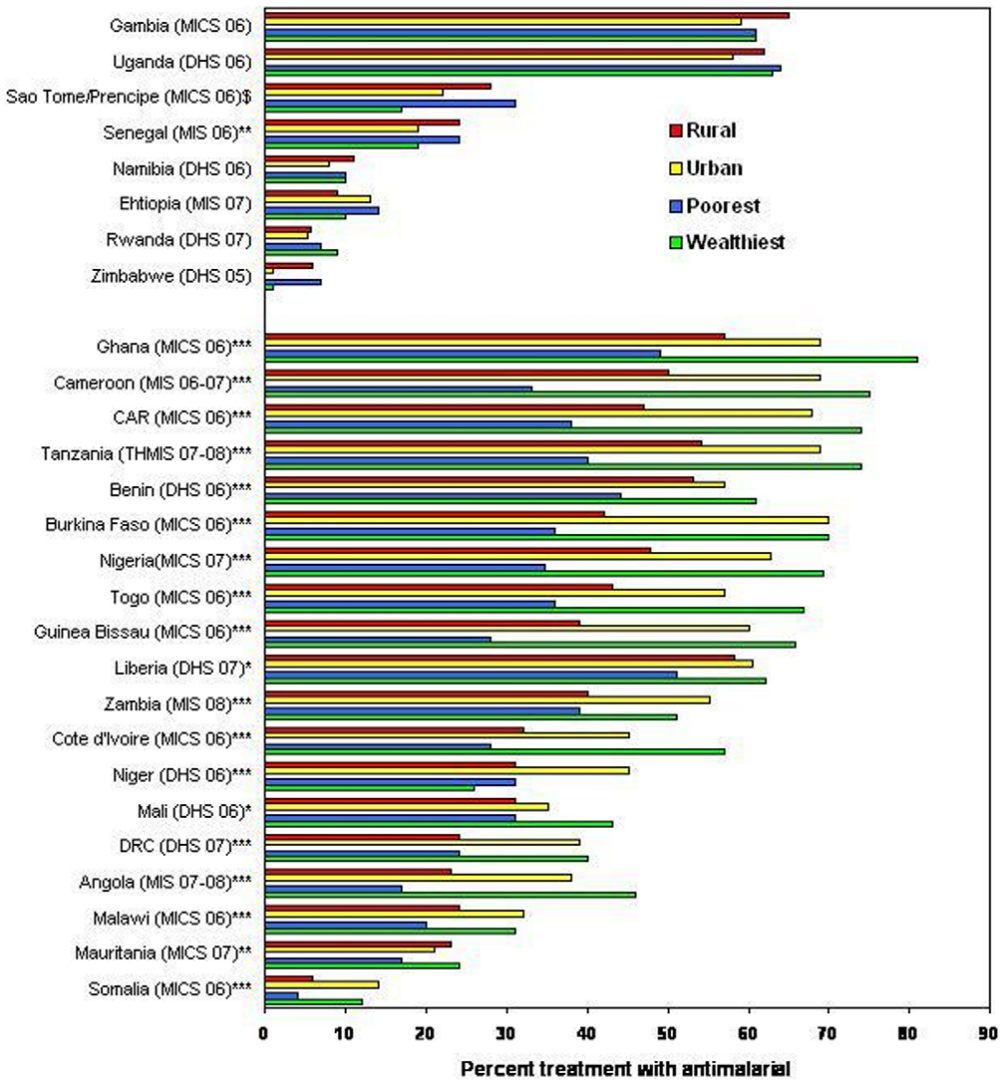
Equity in antimalarial treatment of fever in children. Percent children with a fever in the past 2 weeks receiving any antimalarial, by household residence and poorest versus wealthiest quintile, from national household surveys 2006–2008. Top group of countries are those achieving equity across rural-urban and wealth quintiles; bottom group are those not achieving equity across these categories. *Wealth statistically different (P-value<0.05); **Urban/rural statically different (P-value<0.05); ***Wealth and urban/rural statistically different (P-value<0.05); $Data not available for statistical test.

**Figure 3:**
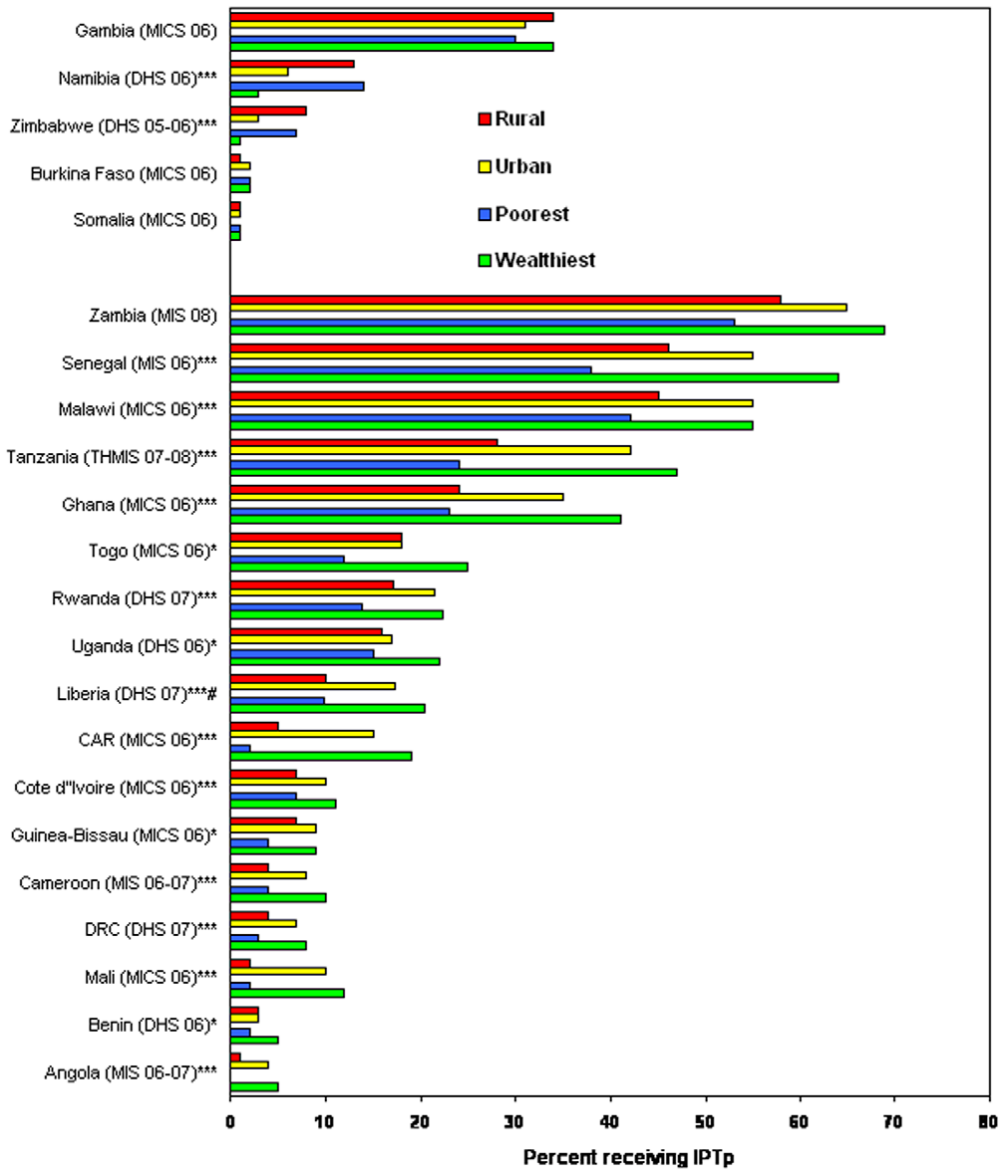
Equity in use of intermittent preventive treatment in pregnancy (IPTp). Percent women 15–49 who received 2 or more doses of sulfadoxine-pyrimethamine for IPTp during their last pregnancy, by rural versus urban residence and poorest versus wealthiest quintile, from national household surveys 2006–2008. Top group of countries are those achieving equity across rural-urban and wealth quintiles; bottom group are those not achieving equity across these categories. *Wealth statistically different (P-value<0.05); **Urban/rural statically different (P-value<0.05); ***Wealth and urban/rural statistically different (P-value<0.05); # Data are for sulfadoxine-pyrimethamine preventive use, but did not specify 2+ doses IPTp.

We identified 27 surveys conducted from 2006 through 2008 with nationally-representative data from malaria-endemic countries in Africa with reports published by July 2009. The country data showed a wide range of intervention coverage (from <10% to >60% population coverage) and the reports systematically presented information on urban and rural settings and on household wealth quintile for most but not all of the interventions. Of note, country coverage levels varied for each intervention and some countries with high coverage for one intervention had relatively low coverage for another intervention: for example, Mali had >50% household ownership of ITNs but <10% coverage with IPTp (Figures 1, 2, 3).

National estimates of household ownership of at least one ITN varied from a high of over 60% in Zambia to approximately 5% in Cameroon (Figure 1). Thirteen (52%) of the 25 countries achieved equitable coverage (richest-to-poorest average equity index = 0.86, [range 0.20 to 1.11]; urban-to-rural average equity index = 0.80, [range 0.34 to 1.19]); see Figure 1, equitable countries in the upper section. There were substantial inequities in the other 12 countries (richest-to-poorest average equity index = 3.27; [range 1.67 to 6.50]; urban-to-rural average equity index = 1.73 [range 1.10 to 3.00]); see Figure 1, lower section.

National coverage estimates of malaria treatment for febrile children under 5 years of age range from 70% in urban Burkina Faso to less than 10% in urban and rural Zimbabwe (Figure 2). Nearly one-third of the countries (8/27) in this analysis achieved coverage that was equitable or favored poor rural households (richest-to-poorest average equity index = 0.81 and urban-to-rural equity index = 0.84); see Figure 2, upper section. Among the 19 countries with inequitable coverage, the greatest disparity is seen by wealth quintile (richest-to-poorest average equity index = 1.81; urban-to-rural average equity index = 1.40) where in five countries the coverage in the poorest households was less than one-half that in the wealthiest households; see Figure 2, lower section.

Population coverage of women receiving IPTp or an antimalarial drug during pregnancy was generally low and exceeded 20% in only 6 (Zambia, Senegal, Malawi, Tanzania, Ghana, and Gambia) of the 22 countries with data available (Figure 3). Although IPTp is meant to reach all women attending antenatal clinic and in many countries the proportion of pregnant women attending antenatal clinic is high, fewer than one-quarter (5/22) of the countries in our analysis achieved equitable or coverage favoring poor rural women; see Figure 3, upper section. In the remaining 17 countries with inequitable coverage, urban women and those in the wealthiest quintile often had a 2-fold or higher coverage compared to rural and poor women; see Figure 3, lower section.

**Figure 4:**
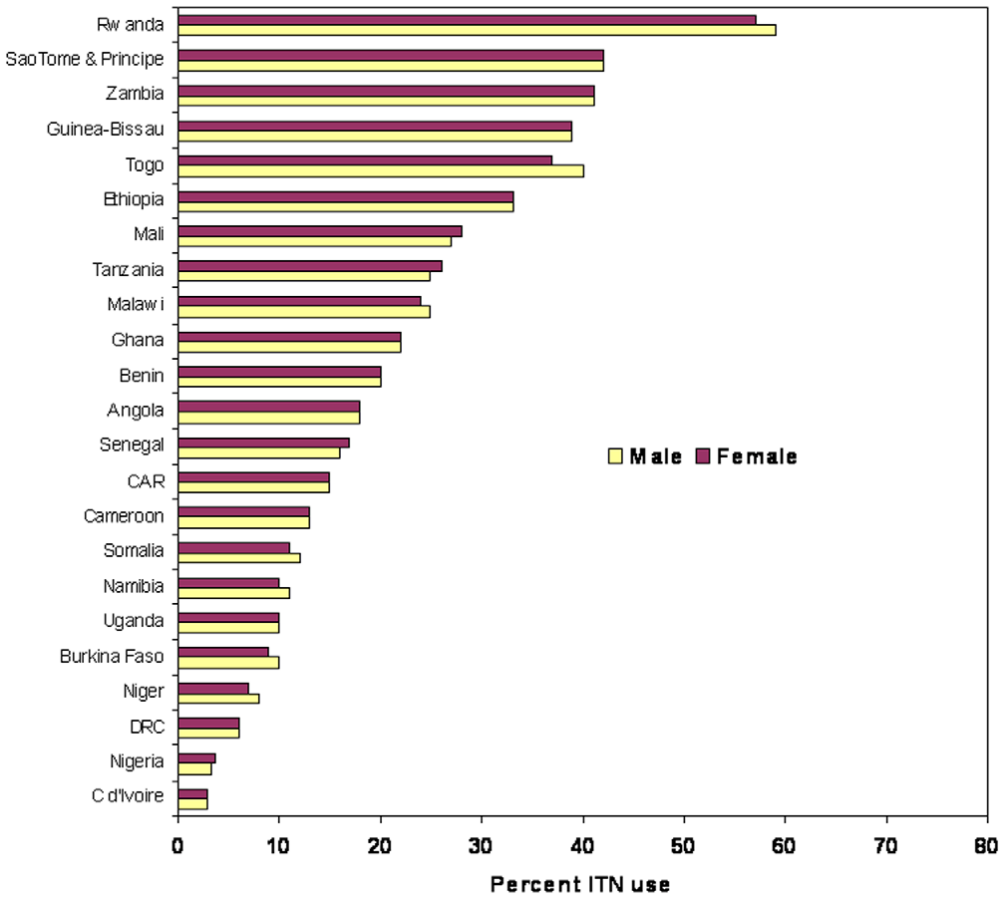
Equity among male and female children sleeping under an ITN. Percent male and female children under-5 years of age sleeping under an ITN the previous night, national surveys between 2006 and 2008. All country male-to-female rates are similar with no statistically significantly differences (P-value>0.05).

**Figure 5:**
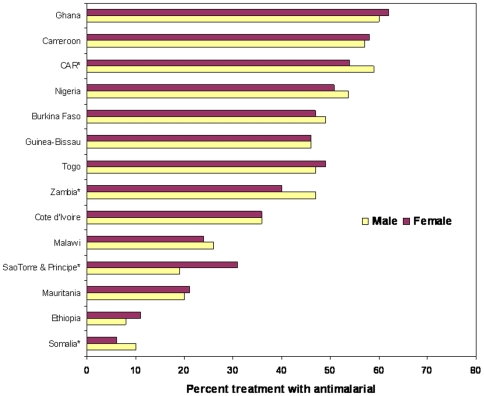
Equity among male and female children receiving antimalarial treatment. Percent of male and female children under-5 years of age with fever receiving any antimalarial medicines, national surveys between 2006 and 2008. *Statically different with P-value<0.05.

No male-female differences were observed among children using an ITN the previous night (23 studies with data) or receiving malaria treatment for fever illness (14 studies with data) (Figures 4 and 5). While overall use levels varied substantially among the countries, there were no survey results showing marked differences between male and female children for ITN use, while only three countries showed significant differences for treatment of fever illnesses; two favoring males and one favoring females.

**Figure 6:**
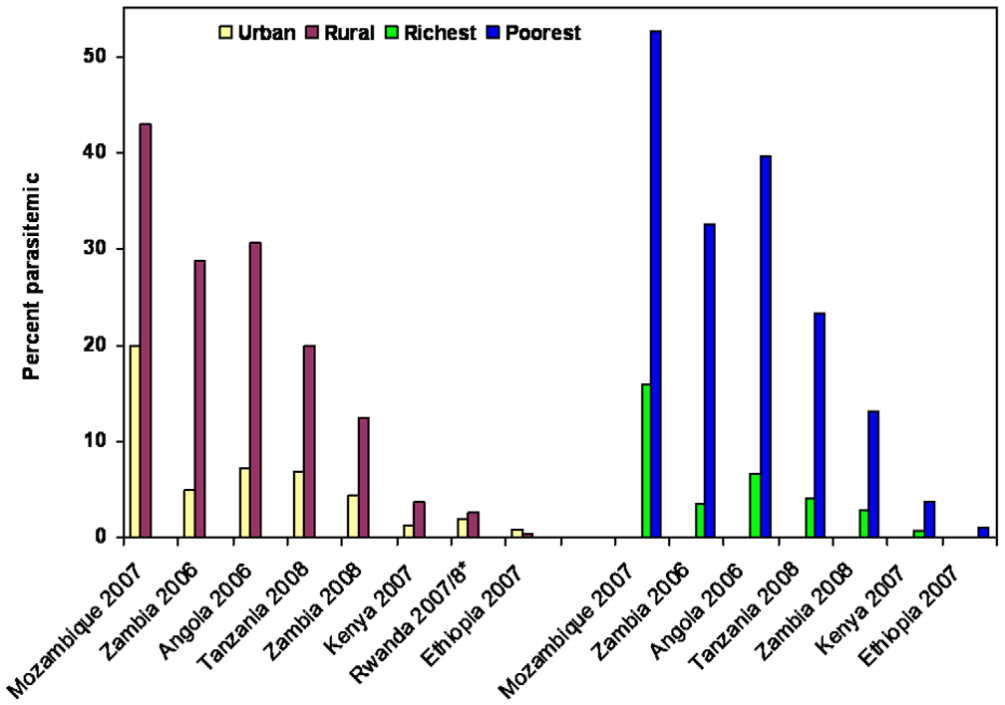
Malaria parasite prevalence in children. Percent parasitemia in children under-5 years of age by urban or rural setting and by richest or poorest wealth quintile, national Malaria Indicator Surveys in African countries. *The Rwanda DHS report did not include parasitemia comparisons by wealth quintile. All urban-rural and richest-poorest differences are statistically significant at P-value<0.001 except for Rwanda urban versus rural (*X^2^* = 1.52, P-value = 0.2168). For Zambia, substantial increases in malaria intervention coverage occurred between the 2006 and 2008 surveys and likely accounts for the observed reduction in prevalence, predominantly in rural and poor populations.

**Figure 7:**
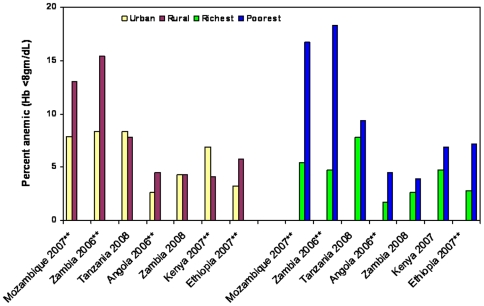
Moderate-to-severe anemia rates in children. Anemia rates (Hb<8gms/dl) in children under-5 years of age by urban or rural setting and by richest or poorest wealth quintile, national Malaria Indicator Surveys in African countries. *While testing was done in the Rwanda DHS, the comparisons were provided in different categories and are not comparable to the other studies. **Statically different with P-value<0.05. For Zambia, substantial increases in malaria intervention coverage occurred between the 2006 and 2008 surveys and likely accounts for the observed reduction in severe anemia, predominantly in rural and poor populations.

Recent data were available on the prevalence of malaria parasitemia and/or moderate-severe anemia (Hb<8 gm/dl) from 8 DHS and MIS [Angola (2006), Ethiopia (2007), Kenya (2007), Mozambique (2007), Rwanda (2007/8), Tanzania (2008) and Zambia (2006 and 2008)]. These findings confirm that malaria and anemia disproportionately affect children in rural and poor households (Figures 6 and 7). Of note, Zambia achieved substantial increases in malaria intervention coverage between the 2006 and 2008 survey and most of the reduction in parasitemia and anemia is seen in the rural and the poor populations.

## Discussion

This analysis of malaria intervention coverage in sub-Saharan Africa shows that many countries have achieved equitable coverage among poor rural households where the burden of malaria is concentrated. The recent progress in malaria control has been sufficiently rapid that we expect that many countries will have both higher and more widely applied intervention coverage in 2009 than might be recorded from these 2006 – 2008 surveys. The analysis also identifies challenges in equitable distribution of interventions that require continued attention as many countries strive to achieve universal coverage for malaria interventions.

Equitable distribution of interventions is possible; 54% of countries have equitable ITN distribution, 29% have equitable case management coverage and 20% have equitable IPTp coverage. But achieving equity in one area does not assure broad achievement of equity; only Namibia and the Gambia achieved equity for all three intervention strategies, and only the Gambia has equitable moderate or high coverage for the interventions.

So, what predicts or determines equity in intervention coverage? While achieving universal coverage will by definition achieve equity, there is no observable dramatic effect whereby countries with higher coverage (50% to 70%) are more likely to have equity at that stage. In fact, across the range from 5% to 70%, coverage per se does not appear from these data to be a critical determinant of equity. At least two factors likely explain the observed inequity in intervention coverage: the policies and choices around delivery strategy used for certain prevention interventions (e.g., the methods used for delivering ITNs, and possibly for IRS) and the existing delivery systems available for providing treatments and the extent of their reach to rural and poor populations (e.g., facility-based and/or community-based services providing treatment for malaria illness or IPTp).

The method of distribution and cost to the end user are critical considerations in achieving equity in ITN coverage [11]. There is growing evidence that equitable household ITN possession is achievable through free wide-scale community distribution [21], [25]-[27]. Partly as a result of such evidence and because ITNs are increasingly viewed as a public good, just like vaccines in children [28], the policies and choices of delivery strategies for ITNs have evolved over the past few years such that there is increasing acceptance that full household population coverage should be sought and any impediments to coverage should be avoided. While it is beyond the scope of this analysis to assess the predominant delivery strategies country by country for the 13 with equitable and 12 with inequitable coverage, it is reasonable to propose that countries with policies that prioritize high household coverage (e.g. an ITN for every sleeping space or one ITN for every two household members), in combination with free wide-scale distribution, will likely improve overall coverage and equity as has been previously observed [12], [21], [29].

For countries using IRS, the spraying is likely to be targeted to certain geographic areas with certain characteristics – typically to urban and peri-urban areas where housing is close together and wall construction materials are amenable to the application of residual insecticides. This targeting may lead to inequity by design, but within these designated areas, the approach of achieving very high coverage (i.e., >90% in the geographically targeted area) should maximize both coverage and equity for that population.

Only two countries (The Gambia and Uganda) reported both high and equitable coverage of treatment of child malaria; and fewer than 50% of febrile children received prompt antimalarial treatment in 14 of the 27 countries studied. In contrast to the variety of delivery methods available for providing prevention commodities (e.g., for ITNs and IRS), it may be more difficult to rapidly achieve equitable coverage of malaria treatment as the largest factor influencing this is overall access to health care. The predominance of infection, illness and severe illness in rural and poor households means that every modality to reach these populations should be pursued. This is not unique to malaria and has been cited as an overarching feature for child health and survival in general [30]. Policies that improve access to health services, such as promoting community outreach and limiting barriers to attending health facilities, will be needed to assure improved and equitable coverage of malaria treatment.

The findings that few countries have achieved more than 20% coverage with IPTp suggests that there remains much work to be done on the initial steps of in-country policy development and delivery strategy with the reproductive health and malaria programs. Given that in many countries a high proportion of pregnant women attend antenatal clinic, with most attending multiple times during pregnancy, there are substantial opportunities for rapid improvement in coverage; and as long as attention is paid to systematically reaching all those who attend, high coverage and good equity may be relatively easy to achieve.

The analysis shows that ITN use and receipt of malaria treatment are equitable among male and female children. The determinants of such equity likely lie principally within the home and are not determined by national policy or health service systems, except perhaps to the extent that they foster a gender-equity message for communities. Mothers and care givers can all be applauded for doing the right thing in all of these countries. Apparently, the only challenge for young boys and girls is that the coverage levels need to rise.

This descriptive assessment of the equity of malaria intervention coverage across countries relied on available national survey reports and relevant analyses. Our analysis did not include assessment of confounding factors; it is clear that wealth and urban-rural dwelling are highly correlated. We also did not adjust our statistical tests for the effect of clustering, which may have biased our results away from the null hypothesis of there being no statistical differences between equity factors. We were also not able to further explore the individual country data to assess additional and potentially important within-country inequities that may exist. For example, “rural” and “urban” categories may hide issues such as “remote rural” versus “rural with access to services”; and wealth quintiles for urban settings may be quite different from wealth quintiles in rural areas. Such additional country-specific analyses do not easily lead to information that can be considered in a multi-country comparison as presented here, but should be considered by individual country programs to further explore their data and their opportunities to expand their program coverage and equity. The goal of this assessment was to examine the extent to which countries have or have not achieved equitable coverage of malaria interventions, defined here as favoring urban and the wealthiest households. For this purpose, the approach used seems adequate as large inequities in coverage clearly exist in some, but not all places.

Equity cannot await universal coverage; it must be programmed at all stages of malaria control scale up. As malaria in Africa is concentrated among children and pregnant women in poor rural areas, the full benefit of malaria control interventions will not be realized unless high coverage among these populations is achieved. Measuring the equity of intervention coverage will remain important in assessing the impact of intervention scale-up on the malaria burden within countries, until universal coverage has been achieved.
